# Docetaxel-carboplatin as first line chemotherapy for epithelial ovarian cancer

**DOI:** 10.1054/bjoc.2000.1572

**Published:** 2001-01

**Authors:** P A Vasey, R Atkinson, R Coleman, M Crawford, M Cruickshank, P Eggleton, D Fleming, J Graham, D Parkin, J Paul, N S Reed, S B Kaye, on behalf of the Scottish Gynaecological Cancer Trials Group

**Affiliations:** CRC Department of Medical Oncology, Beatson Oncology Centre, Western Infirmary, Glasgow, G11 6NT; Belfast City Hospital, Belfast BT9 7AB, Belfast; YCR Cancer Research Centre, Weston Park Hospital, Sheffield, S10 2SJ; Airedale General Hospital, Steeton, West Yorkshire, BD20 6TD; Aberdeen Royal Infirmary, Aberdeen, AB9 2ZB; Rhone-Poulenc (now Aventis Pharma), Anthony, France; Bristol Oncology Centre, Bristol, BS2 8ED

**Keywords:** ovarian carcinoma, chemotherapy, docetaxel, carboplatin

## Abstract

A prospective, non-randomized, multicentre, open, dose-finding study of a carboplatin-docetaxel (C-D) combination as first-line chemotherapy in FIGO stage Ic–IV epithelial ovarian cancer. C-D was given 3-weekly for 6 planned cycles, with a 3-day prophylactic dexamethasone regimen (8 mg b.i.d.). 139 eligible patients (Pts) (median age 56 years, range 28–85) were given a total of 750 cycles of chemotherapy in 5 cohorts: Co1, 32 pts, 169 cycles (C at AUC 5 + D 60 mg/m^2^); Co2, 22 pts, 122 cycles (5 + 75), Co3, 29 pts, 156 cycles (6 + 75), Co4, 27 pts, 146 cycles (7 + 75), Co5, 30 pts, 157 cycles (6 + 85). 110 patients (79%) completed 6 cycles; 17 (12%) stopped due to toxicity. 104 patients (75%) had CTC grade IV neutropenia, and 5 patients (4%) had this associated with fever. There were 2 probable treatment-related deaths. Only 8 patients (6%) experienced grade II–III neurotoxicity (all sensory; no motor > grade I). The maximum tolerated dose was reached in cohorts 4 and 5, and the dose limiting toxicities were myelosuppression and diarrhoea. The overall response rate for the study was 66% (49/74); CA125 response was 75% (70/93). Median progression-free survival was 16.6 months (95% CI 13.3–19.1). Recommended doses are carboplatin AUC 5 (via^51^ Cr EDTA) or AUC 6 (if calculated) plus docetaxel 75 mg/m^2^. A randomized trial comparing this regimen with carboplatin-paclitaxel has just completed recruitment. © 2001 Cancer Research Campaign http://www.bjcancer.com


*British Journal of Cancer* (2001) **84**(2), 170–178
© 2001 Cancer Research Campaign
doi: 10.1054/ bjoc.2000.1572, available online at http://www.idealibrary.com on IDEAL®

http://www.bjcancer.com

### Docetaxel-carboplatin as first line chemotherapy for epithelial ovarian cancer

**PA Vasey[1], R Atkinson[2], R Coleman[3], M Crawford[4], M Cruickshank[5], P Eggleton[6], D Fleming[1], J Graham[7], D Parkin[5], J Paul[1], NS Reed[1] and SB Kaye[1] on behalf of the Scottish Gynaecological Cancer Trials Group**

[1]CRC Department of Medical Oncology, Beatson Oncology Centre, Western Infirmary, Glasgow, G11 6NT; [2]Belfast City Hospital, Belfast, Belfast BT9 7AB; [3]YCR Cancer Research Centre, Weston Park Hospital, Sheffield, S10 2SJ; [4]Airedale General Hospital, Steeton, West Yorkshire, BD20 6TD; [5]Aberdeen Royal Infirmary, Aberdeen AB9 2ZB; [6]Rhone-Poulenc (now Aventis Pharma), Anthony, France*; [7]Bristol Oncology Centre, Bristol, BS2 8ED

**Summary** A prospective, non-randomized, multicentre, open, dose-finding study of a carboplatin-docetaxel (C-D) combination as first-line chemotherapy in FIGO stage Ic–IV epithelial ovarian cancer. C-D was given 3-weekly for 6 planned cycles, with a 3-day prophylactic dexamethasone regimen (8 mg b.i.d.). 139 eligible patients (Pts) (median age 56 years, range 28–85) were given a total of 750 cycles of chemotherapy in 5 cohorts: Co1, 32 pts, 169 cycles (C at AUC 5 + D 60 mg/m$^2$); Co2, 22 pts, 122 cycles (5 + 75), Co3, 29 pts, 156 cycles (6 + 75), Co4, 27 pts, 146 cycles (7 + 75), Co5, 30 pts, 157 cycles (6 + 85). 110 patients (79%) completed 6 cycles; 17 (12%) stopped due to toxicity. 104 patients (75%) had CTC grade IV neutropenia, and 5 patients (4%) had this associated with fever. There were 2 probable treatment-related deaths. Only 8 patients (6%) experienced grade II–III neurotoxicity (all sensory; no motor > grade I). The maximum tolerated dose was reached in cohorts 4 and 5, and the dose limiting toxicities were myelosuppression and diarrhoea. The overall response rate for the study was 66% (49/74); CA125 response was 75% (70/93). Median progression-free survival was 16.6 months (95% CI 13.3–19.1). Recommended doses are carboplatin AUC 5 (via $^{51}$Cr EDTA) or AUC 6 (if calculated) plus docetaxel 75 mg/m$^2$. A randomized trial comparing this regimen with carboplatin-paclitaxel has just completed recruitment. © 2001 Cancer Research Campaign http://www.bjcancer.com

**Keywords**: ovarian carcinoma; chemotherapy; docetaxel; carboplatin

Epithelial carcinoma of the ovary is one of the most common gynaecologic malignancies, and the fourth most frequent cause of cancer death in women (Yancik, 1993). The asymptomatic early stages of ovarian cancer mean that most patients have widespread disease at the time of diagnosis. Patients with FIGO stage III and IV disease and significant residual tumour masses after primary surgery can expect a 5-year survival rate of less than 10%, despite multiple courses of platinum-based chemotherapy (Omura et al, 1991).

Until the mid-1990s, the combination of a platinum compound and an alkylating agent was considered best therapy for these patients, but a new standard of care emerged after the publication of trial GOG-111 in 1996 (McGuire et al, 1996) which was followed by the first reports of the INTERGROUP trial, OV10, in 1998 (Piccart et al, 2000). Both these large, prospective randomized studies demonstrated that patients treated with cisplatin-paclitaxel in combination had significantly higher response rates, progression-free survival and overall survival compared with the previous standard treatment of cisplatin and cyclophosphamide. However, this higher efficacy was at the expense of greater toxicity. In GOG-111, 135 mg/m$^2$ paclitaxel was administered with 75 mg/m$^2$ cisplatin as an inconvenient 24-hour infusion, and neurotoxicity in particular was more frequently observed (28% vs 21%, $P \leq 0.05$). Reducing the paclitaxel infusion time to 3 hours (with a concomitant increase in dose to 175 mg/m$^2$) in OV.10 produced a further increase in neurotoxicity, with 19.6% patients experiencing CTC grade III or IV sensory neurotoxicity, compared with 1% of patients receiving cisplatin-cyclophosphamide.

Meta-analyses incorporating data on nearly 10 000 patients from 45 randomized trials suggested that the substitution of carboplatin for cisplatin was equally effective either as a single agent or in combination (Aabo et al, 1998). The addition of carboplatin to paclitaxel was expected to produce less emesis and neurotoxicity, but greater myelosuppression compared with cisplatin-paclitaxel. Seven phase I–II trials of carboplatin-paclitaxel combinations have been reported, involving 260 chemo-naïve ovarian cancer patients (Bookman et al, 1996; Lhomme et al, 1996; Bolis et al, 1997; duBois et al, 1997; Huizing et al, 1997; ten Bokkel Huinink et al, 1997; Siddiqui et al, 1997). Doses of carboplatin ranged from AUC 5–10, and paclitaxel from 120–250 mg/m$^2$ and almost all the trials used a 3-hour paclitaxel administration schedule. As expected the major toxicities in all studies were myelosuppression and neurotoxicity, however, an apparent reduction in the expected level of thrombocytopenia was observed in many of these trials, and an interaction at the megakaryocyte level rather than a pharmacokinetic interaction is thought to be responsible (Calvert et al, 1995). Antitumour activity was substantial, with response rates ranging from 70–100%.

The direct comparison of carboplatin-paclitaxel with cisplatin-paclitaxel in a prospective, randomized trial as first-line therapy for advanced ovarian cancer has now been the subject of three phase III trials (Neijt et al, 1997; duBois et al, 1999; Ozols et al, 1999).

*Received 8 June 2000*
*Revised 21 September 2000*
*Accepted 11 October 2000*

*Correspondence to:* PA Vasey

*Current address: Merck KGaA, Darmstadt, Germany

The Danish–Dutch study (Neijt et al, 1997) randomized 208 patients with stage IIB–IV disease to receive paclitaxel 175 mg/m$^2$ over 3 hours in combination with either carboplatin AUC 5 or cisplatin 75 mg/m$^2$. Although this study had relatively few patients, it suggested that the carboplatin arm was at least as effective as cisplatin, with better patient tolerance. The level of neurotoxicity was reported to be similar in the two arms; only 26% (carboplatin) and 18% (cisplatin) patients reported no neurologic symptoms during their chemotherapy. The treatments appeared to be equally active, with response rates of 70% and 73% respectively for the carboplatin and cisplatin combinations, and median progression-free survivals of 17 months in both arms.

Patients in GOG trial 158 differed from the Danish–Dutch study in that only optimally debulked (to <1 cm residual tumour) stage III patients were eligible. The reason for this protocol design was based on concerns from in vitro data and clinical trials in other tumour types (i.e. testicular germ cell tumours) which suggested that the substitution of carboplatin for cisplatin for patients with potentially curable disease could be detrimental to their outlook. The trial was therefore designed with the hypothesis that changing cisplatin for carboplatin would decrease the progression free survival in this potentially curable patient group. Hence, the choice of the control arm as per the GOG 111 cisplatin-paclitaxel schedule, versus a study arm of carboplatin AUC 7.5 in combination with paclitaxel 175 mg/m$^2$ over 3 hours. With 425 and 415 patients randomized to each arm respectively, and a median follow up of 19 months, the first survival analysis reported no difference in efficacy (Ozols et al, 1999). A median time to progression of 22 months was observed for each patient group, and although more mature data will be required for confirmation, the survival curves are unlikely to deviate significantly. As expected, differences in toxicity between the two treatment arms were apparent, with more haematologic toxicity observed for carboplatin, and more non-haematologic toxicity for cisplatin. However, no differences were observed with respect to the incidence of clinically significant neurotoxicity.

Finally, the AGO group trial OVAR-3 randomized patients with stage IIB–IV disease to receive either cisplatin 75 mg/m$^2$ or carboplatin AUC 6 in combination with paclitaxel 185 mg/m$^2$, regardless of tumour bulk Paclitaxel was administered over 3 hours in both arms. After the first analysis with 392 and 384 patients randomized, there was no significant difference in median progression-free survival between the treatments (73 and 69 weeks respectively), and this equivalence remains when patients are stratified as either ‘good risk’ (<1 cm, IIB–III) or ‘poor risk’ (>1 cm, III/IV) (duBois et al, 1999). Once again, the expected toxicity differences were noted, but here, in contradistinction to GOG 158, there was more neurotoxicity observed in the cisplatin arm than the carboplatin arm (grade III/IV, 19% vs 8%).

Together, these trials suggest that the more convenient carboplatin-paclitaxel combination is generally better tolerated than cisplatin-paclitaxel and appears to be equally efficacious. However, some concerns remain. The 3-hour infusion of paclitaxel in combination with carboplatin is still associated with significant neurotoxicity, and there is still the possibility based on in vitro data that longer schedules of paclitaxel may be somewhat more efficacious as first line therapy (Gianni, 1995).

Docetaxel (Taxotere®) has demonstrated single-agent efficacy at least equivalent to paclitaxel, with an overall response rate of 28% in 155 platinum-refractory ovarian cancer patients (Kaye et al, 1997). Also, clinical studies in breast cancer have confirmed previous in vitro observations which described incomplete cross-resistance with paclitaxel (Valero, 1996), and randomized trials in breast cancer indicate superiority of docetaxel over doxorubicin, while this has not been seen for paclitaxel (Chan et al, 1997; Paridaens et al, 1997). There is preliminary evidence of activity in ovarian cancer patients who have failed prior paclitaxel (Kavanagh et al, 1999). Moreover, docetaxel is generally delivered as a convenient 1-hour infusion, suitable for out-patient administration.

The Scottish Gynaecological Cancer Trials Group (SGCTG) have previously carried out a prospective, non-randomized feasibility study of a cisplatin-docetaxel combination in 100 chemonaïve stage Ic–IV ovarian cancer patients (Vasey et al, 1999). Doses of 75 mg/m$^2$ of both agents in combination appeared to be feasible, and the delivery of multiple cycles of docetaxel was not abrogated by fluid retention. However, patient tolerance was relatively poor. Grade III/IV neutropenia was observed in more than 75% patients and appeared to be cumulative, 33% patients were unable to complete the planned 6 cycles, and increasing the dose of docetaxel to 85 mg/m$^2$ produced unacceptable haematologic toxicity and increased risk of morbidity. A response rate of 69% was observed, but the median progression-free survival for the group was only 12 months. This may in part be explained by the poor treatment completion rate due to toxicity.

The ability of carboplatin to decrease the treatment-related toxicity in combination with paclitaxel with no loss of efficacy, led the SGCTG to initiate a prospective, non-randomized, feasibility study of docetaxel-carboplatin as first-line therapy for ovarian cancer patients. The aim was to establish whether patterns of toxicity differed from those experienced with paclitaxel-carboplatin, with particular reference to myelotoxicity and neurotoxicity. A favourable outcome would be expected to lead to a subsequent randomized trial.

### METHODS

### Patients

Eligible women had histologically verified epithelial ovarian cancer, were over 18 years old and had FIGO stages Ic–IV with or without successful cytoreductive surgery at staging laparotomy. Stage Ic disease was limited to patients with malignant cells in ascitic fluid or peritoneal washings, pre-operative capsular rupture or surface tumour. Patients had an ECOG performance status of ≤ 2, and adequate bone marrow, renal and hepatic function. Written, informed consent in compliance with the recommendations of the Declaration of Helsinki was obtained in all cases.

Patients were ineligible for study entry if they had any prior treatment with chemotherapy or radiotherapy, or any prior malignancy (except for curatively treated carcinoma in situ of the uterine cervix or basal cell carcinoma of the skin). Borderline ovarian tumours or abdominal adenocarcinoma of unknown origin were excluded, as were patients with clinically significant pleural effusions or ascites unless confirmed cytologically to be due to ovarian cancer. Patients were also ineligible if there was a history of medically significant atrial or ventricular dysrhythmias, congestive heart failure, or documented myocardial infarction within the 6 months preceding study entry. Additional contraindications included; active infection or serious intercurrent illness that was judged by the investigators likely to impair the patients’ ability to receive protocol therapy; a history of prior serious allergic

© 2001 Cancer Research Campaign reactions; symptomatic peripheral neuropathy >grade I. Pregnant or lactating women were ineligible, but potentially fertile women using adequate contraception were allowed treatment. No patients with insulin-dependent diabetes mellitus were enrolled.

### Treatment plan and administration

Docetaxel 60–85 mg/m$^2$ and carboplatin AUC 5–7 were administered consecutively on day 1 of a 21 day cycle, for 6 planned cycles. Carboplatin was dosed according to the Calvert formula (glomerular filtration rate + 25)  desired AUC (Calvert et al, 1989), where the glomerular filtration rate was measured by $^{51}$CrEDTA (Chantler et al, 1969). This dose remained fixed throughout subsequent cycles, unless de-escalation was required due to toxicity. Patients who had either a partial response or stable disease after 6 cycles were allowed to receive further chemotherapy with 3 cycles of single agent carboplatin, AUC 5–7 depending on the clinician's preference. The appropriateness of either second look or interval cytoreductive surgery was determined on an individual patient basis, as this was not a protocol requirement. The 5 treatment cohorts are described in Table 1. Patients were entered into each dose cohort until the first 6 had completed 2 full treatment cycles. Escalation to the next dose cohort proceeded if <2 of these 6 patients (and <33% of any other patients concurrently receiving chemotherapy in that dose cohort) developed dose-limiting toxicity (DLT). The Maximum Tolerated Dose (MTD) was defined as the dose level at which ≥2 of the first 6 patients followed up for 2 complete cycles (or ≥33% of the patients currently receiving chemotherapy at this dose level) experienced either (a) complicated or prolonged grade IV neutropenia, (b) complicated grade IV thrombocytopenia and/or requiring platelet transfusion, (c) any grade III non-haematologic toxicity excluding emesis and alopecia.

Premedication consisted of oral dexamethasone 8 mg b.i.d. for 3 days starting the day before chemotherapy. Docetaxel was reconstituted in 250 ml of 5% glucose and administered by intravenous infusion over 60 minutes. Carboplatin was then administered in 500 ml of 5% glucose over 30–60 minutes. Prophylactic intravenous antiemetics (8 mg dexamethasone plus either 3 mg granisetron (Kytril®, SmithKline Beecham Pharmaceuticals, Surrey, UK) or 8 mg ondansetron (Zofran®, Glaxo Wellcome Ltd, Middlesex, UK)) were administered to all patients immediately prior to the docetaxel infusion. All patients were routinely prescribed oral domperidone (Motilium®, Sanofi Winthrop Ltd, Surrey, UK) 20 mg t.i.d.-q.i.d. as required for 5–7 days following chemotherapy.

**Table 1** Dose cohorts and treatment delivery

| Cohort | Carboplatin (AUC) | Docetaxel (mg/m$^2$) | No. patients registered | No. cycles | Completed 6 cycles |
|---|---|---|---|---|---|
| 1 | 5 | 60 | 32 | 169 | 24 (75%) |
| 2 | 5 | 75 | 22 | 122 | 20 (91%) |
| 3 | 6 | 75 | 29[a] | 156 | 23 (82%) |
| 4 | 7 | 75 | 28[b] | 146 | 21 (78%) |
| 5 | 6 | 85 | 30 | 157 | 22 (73%) |

[a]1 patient in this cohort refused study treatment before starting and has been excluded from all subsequent analyses. [b]1 patient in this cohort was ineligible (wrong stage) and has been excluded from all subsequent analyses.

### Dose/schedule modifications

Treatment was administered on d1 of each planned 21 day cycle if the neutrophils were ≥1.5 × 10$^9$ l$^{-1}$ and platelets ≥100 × 10$^9$ l$^{-1}$; values less than this necessitated a treatment delay until recovery. Any delay more than 2 weeks for haematological recovery meant termination of protocol therapy. Dose reductions were based upon nadir blood counts. Any grade IV neutropenia that lasted at least 7 days and/or was complicated by fever resulted in a reduction of docetaxel by 10–15 mg/m$^2$ on all subsequent cycles. Any such neutropenic events were treated at the time with antibiotics and G-CSF was added if considered appropriate by the investigator. The occurrence of neutropenic fever also resulted in prophylactic oral antibiotics (ciprofloxacin 250 mg b.i.d., day 5–15) being prescribed for each subsequent treatment cycle. If complicated or prolonged neutropenia occurred again, despite dose reductions and prophylactic antibiotics, subsequent cycles were delivered with subcutaneous G-CSF 300 μg d$^{-1}$ from d5–14 or until the neutrophil count was > 1.0 × 10$^9$ and rising. Grade IV thrombocytopenia requiring platelet transfusion and/or complicated by haemorrhage resulted in a reduction of the carboplatin dose by 10% in all subsequent cycles

Abnormalities of hepatic function as evidenced by aminotransferase (AST/ALT) and/or alkaline phosphatase (ALP) elevations to > grade I during treatment, resulted in the patient being withdrawn from protocol therapy, and were treated with carboplatin as a single agent.

Treatment delays were planned for patients who developed severe skin toxicity (≥ grade III) for a maximum 2 weeks until recovery to ≤ grade I, when they could be re-treated with a 10–15 mg/m$^2$ reduction of docetaxel. Mucositis ≥ grade II necessitated a treatment delay of maximum 2 weeks until resolution of lesions, and a subsequent docetaxel dose reduction as above. No dose reductions were planned on the basis of docetaxel-induced fluid retention. The development of grade III/IV neurotoxicity – motor, sensory or otologic – necessitated termination of protocol therapy.

Mild hypersensitivity reactions were treated by slowing down the docetaxel infusion. Severe hypersensitivity reactions were terminated with appropriate drug therapy (adrenaline, antihistamines, corticosteroids, depending upon the severity). Rechallenge after recovery from a hypersensitivity reaction was allowed if clinically indicated, and was generally done within 3 hours. Later rechallenges (3–24 hours) were required to be further premedicated with high dose dexamethasone and chlorpheniramine (Piriton®, Stafford-Miller Ltd, Herts, UK). Further hypersensitivity reactions necessitated withdrawal from study.

### Patient evaluation and clinical assessments

Patients underwent full physical examination including vaginal/rectal examination. Baseline investigations prior to study entry included; full blood count and differential white cell count, biochemical profile (including urea, creatinine, sodium, potassium, calcium, magnesium, AST, ALT, ALP, bilirubin, total protein, albumen, glucose), CA125, $^{51}$CrEDTA measurement of glomerular filtration rate, chest X-ray, and 12-lead electrocardiogram. The size and extent of residual disease was documented by CT scan of abdomen and pelvis. Patients' weight and ECOG performance status were noted at baseline.

During chemotherapy, patients were seen weekly for full blood count, serum chemistry and documentation of treatment-related

*© 2001 Cancer Research Campaign*

toxicity using the National Cancer Institute of Canada Expanded Common Toxicity Criteria (NCIC-CTC). Prior to each treatment cycle, patients were weighed and had a full physical examination plus CA125 estimations. Response to chemotherapy was assessed after 3 and 6 (and if appropriate, 9) courses of chemotherapy by the same imaging technique used at baseline. Clinical and radiological tumour response was graded according to standard criteria (Miller et al, 1981); CA125 responses were graded according to the schema from Rustin (Rustin et al, 1996).

Following completion of protocol chemotherapy, patients were followed up 2-monthly for the first 2 years, 4-monthly to 5 years and annually thereafter. Pelvic examination was carried out at each follow-up visit, along with CA125 measurement. CT scans were carried out if progressive disease was clinically suspected or CA125 levels began to increase.

### Statistical methods

Analysis of variance (Anova) techniques were used to compare cycle 1 nadir neutrophil and platelet counts between cohorts (after suitable transformations to make the data approximately Normal). Similarly cumulative haematological toxicity was examined using repeated measures Anova. Proportions were compared using Pearson's chi-square test (unadjusted). All survival times are taken from the date the patient was registered onto the study. Progression-free survival is the time from registration to progression or death (from any cause). Survival curves were determined using Kaplan-Meier estimates.

### RESULTS

### Patient characteristics and treatment summary

Between 16 April 1997 and 18 March 1998, 141 patients were enrolled into this trial by 9 institutions of the Scottish Gynaecological Cancer Trials Group. One patient in cohort 4 was wrongly staged and received no treatment, and one patient in cohort 3 refused study treatment before starting cycle 1. These 2 patients have been excluded from the remainder of the analysis. Pretreatment characteristics by dose cohort are shown in Table 2. All patients had ovarian epithelial adenocarcinoma (serous adenocarcinoma 94 patients/67%; endometrioid carcinoma 21 patients/15%; mucinous adenocarcinoma 9 patients/6%; clear cell carcinoma 7 patients/5%; others 9 patients/6%). Overall, the median age was 56 years (range 28–85), 110 patients (79%) were FIGO stage III/IV at presentation, and 124 (89%) were performance status 0–1. There were approximately equal numbers of optimally (74 patients/54%) vs suboptimally (62/46%) debulked patients in the group as a whole. In addition, 17 patients had further surgery; 13 patients had secondary cytoreductive surgery (8 patients after 6 cycles, 4 after 3, 1 after 9), and 5 patients underwent a 'second look' laparotomy following 6 cycles. One patient had both secondary cytoreductive surgery and a second look laparotomy.

### Toxicity summary

Overall, 750 cycles of docetaxel-carboplatin chemotherapy were delivered to 139 eligible patients in 5 dose cohorts. 110 patients received all 6 planned cycles of docetaxel and carboplatin (completion rate 79%), and only 17 patients (12%) came off protocol therapy early because of toxicity.

Haematologic toxicity was observed in all treatment cohorts, and is presented in Table 3. 104 patients (75%) developed grade IV neutropenia during the course of their treatment, and the incidence of grade IV neutropenia and associated neutropenic problems by treatment cohort is presented in Table 4. The incidence of grade IV neutropenia ranged from 47% in cohort 1 to 93% in cohort 5, and was significantly less in cohort 1 compared with the other cohorts combined ($P < 0.001$). In addition, there was a linear downward trend for cycle 1 neutrophil nadir counts over cohorts 1–4 ($P = 0.014$) or cohorts 1–3 and 5 ($P = 0.005$). Although the incidence of grade IV neutropenia was high, there were few episodes of febrile neutropenia (5 patients/4%) documented. One patient in cohort 4 died with a grade IV neutropenia and unspecified infection during her second cycle of chemotherapy. Another patient died suddenly at home on d8 following her first cycle on cohort 5. Unfortunately no blood counts or post mortem examinations were carried out, and the patient was therefore classified as a probable toxic death. 22 patients (16%), required dose delays at the beginning of a

**Table 2** Pretreatment characteristics

| | | Cohort 1 ($n$ = 32) Col% | $n$ | Cohort 2 ($n$ = 22) Col% | $n$ | Cohort 3 ($n$ = 28) Col% | $n$ | Cohort 4 ($n$ = 27) Col% | $n$ | Cohort 5[a] ($n$ = 30) Col% | $n$ |
|---|---|---|---|---|---|---|---|---|---|---|---|
| Perf. Status | 0 | 34% | 11 | 45% | 10 | 20% | 7 | 33% | 9 | 62% | 18 |
| | 1 | 59% | 19 | 45% | 10 | 64% | 18 | 48% | 13 | 31% | 9 |
| | 2 | 3% | 1 | 5% | 1 | 11% | 3 | 19% | 5 | 7% | 2 |
| | 3 | 3% | 1 | 5% | 1 | 0% | 0 | 0% | 0 | 0% | 0 |
| Residual disease[b] | ≤ 2 cm | 52% | 16 | 52% | 11 | 63% | 17 | 41% | 11 | 63% | 19 |
| | > 2 cm | 46% | 15 | 48% | 10 | 37% | 10 | 59% | 16 | 37% | 11 |
| Stage | Ic | 3% | 1 | 0% | 0 | 14% | 4 | 7% | 2 | 17% | 5 |
| | II | 6% | 2 | 18% | 4 | 7% | 2 | 22% | 6 | 7% | 2 |
| | III | 72% | 23 | 82% | 18 | 68% | 19 | 59% | 16 | 72% | 21 |
| | IV | 19% | 6 | 0% | 0 | 11% | 3 | 11% | 3 | 3% | 1 |
| Age | Median | 56 | | 50 | | 57 | | 56 | | 55 | |
| | IQ range | 48–61 | | 46–59 | | 53–63 | | 49–62 | | 49–60 | |
| | Range | 32–71 | | 28–74 | | 43–69 | | 28–70 | | 33–85 | |

[a] Data not returned on performance status and stage for patient 129 (cohort 5). [b] Data not available on 3 patients (2 in cohort 1, 1 in cohort 3).

© 2001 Cancer Research Campaign 

**Table 3** Haematological toxicity (worst grade over cycles received)

| | Grade | Cohort 1 (*n* = 32) Col% | *n* | Cohort 2 (*n* = 22) Col% | *n* | Cohort 3 (*n* = 28) Col% | *n* | Cohort 4 (*n* = 27) Col% | *n* | Cohort 5 (*n* = 30) Col% | *n* |
|---|---|---|---|---|---|---|---|---|---|---|---|
| Neutropenia | 3 | 28% | 9 | 9% | 2 | 21% | 6 | 15% | 4 | 7% | 2 |
| | 4 | 47% | 15 | 77% | 17 | 75% | 21 | 82% | 22 | 93% | 28 |
| Leucopenia | 3 | 44% | 14 | 59% | 13 | 54% | 15 | 56% | 15 | 63% | 19 |
| | 4 | 0% | 0 | 5% | 1 | 14% | 4 | 22% | 6 | 17% | 5 |
| Thrombocytopenia | 3 | 9% | 3 | 14% | 3 | 14% | 4 | 30% | 8 | 23% | 7 |
| | 4 | 0% | 0 | 0% | 0 | 7% | 2 | 11% | 3 | 3% | 1 |
| Anaemia | 3 | 19% | 6 | 9% | 2 | 18% | 5 | 26% | 7 | 13% | 4 |
| | 4 | 0% | 0 | 5% | 1 | 0% | 0 | 4% | 1 | 0% | 0 |

**Table 4** Grade IV/complicated neutropenia

| | | Co1 | Co2 | Co3 | Co4 | Co5 | Total |
|---|---|---|---|---|---|---|---|
| Patients | | 32 | 22 | 28 | 27 | 30 | 139 |
| Cycles | | 169 | 122 | 156 | 146 | 157 | 750 |
| Grade IV (overall) | | 15 (47%) | 17 (77%) | 21 (75%) | 22 (82%) | 28 (93%) | 103 (74%) |
| prolonged >7d | gd IV | 2 (6%) | 3 (14%) | 2 (7%) | 5 (19%) | 1 (3%) | 13 (9%) |
| with fever | gd IV | 0 | 1 (3%) | 2 (7%) | 0 | 2 (7%) | 5 (5%) |
| dose reductions | gd IV | 1 (6%) | 1 (6%) | 2 (10%) | 3 (14%) | 1 (4%) | 8 (8%) |

treatment cycle as a direct result of unresolved toxicity. There was evidence of cumulative neutropenia over the first 3 cycles of chemotherapy in all cohorts with the nadir value of cycle 3 around 40% less than that on cycle 1 ($P < 0.001$, test for linear trend over cycles 1–3). On cycles 4–6 nadir levels are roughly consistent.

Evidence for a platelet-sparing effect was noted for docetaxel-carboplatin. Despite $^{51}$CrEDTA-measured carboplatin AUCs of 5–7, Table 3 demonstrates that only 6 patients (4.2%) developed grade IV thrombocytopenia during the course of their treatment. 5 patients received prophylactic platelet transfusions (1 in cohort 3, 4 in cohort 4), as this was left to the policy of the individual hospital. No thrombocytopenic haemorrhages were reported, although one patient experienced a minor bleed from her colostomy and received a platelet transfusion. Cohort 4 (carboplatin AUC 7) cycle 1 nadir platelet counts were found to be significantly less than cohorts 1–3 ($P < 0.05$ for each pairwise comparison after Bonferroni adjustment). A substantial drop in the average nadir platelet count of $50 \times 10^9$ $1^{-1}$ was demonstrated in all cohorts between cycles 1 and 2 ($P < 0.001$). Thereafter, the nadir count only decreased by around $20 \times 10^9 1^{-1}$ between cycles 2 and 5 ($P < 0.001$, test for linear trend over cycles 2–5) and actually appeared to slightly increase on cycle 6. Overall, the incidence of grade IV thrombocytopenia was not statistically different between all treatment cohorts ($P = 0.19$).

Anaemia was commonly observed, but usually not at significant levels (grade III, 24 patients/17%; grade IV, 2 patients/1%). The decision to transfuse packed cells was left to the individual investigator, and was based upon clinical symptoms in addition to the haemaglobin level. Only 3 cycles of chemotherapy (0.4%; all in cohort 5) were delayed because of significant anaemia.

Significant non-haematologic toxicity was uncommon, and overall the combination was well tolerated. Fatigue or lethargy during treatment was reported by over 50% patients, but only 5 patients reported this non-specific symptom at grade III. There were no statistically significant differences in non-haematologic toxicity between the 5 treatment cohorts, and this is shown in Table 5.

Significant emesis was rare; no patients experienced grade IV nausea or vomiting, and only 6% (nausea) and 4% (vomiting) experienced grade III. Severe diarrhoea was observed in only 4 patients, although because of the definition of MTD, this was one of the toxicities that described the DLT in cohorts 4 and 5. Constipation was more common, but again only 2 patients reported grade III toxicity. The routine use of the antiemetic granisetron (Kytril™) may have contributed to this symptom. Grade III mucositis was reported by only 1 patient.

The incidence of significant neurotoxicity was especially low. Table 5 describes the incidence of all grades of motor and sensorineural neurotoxicity during the study. Overall, 36 patients (26%) experienced treatment-related peripheral neuropathy during the study. This was defined in 28 patients (20%) as grade I, in only 7 patients at grade II (4.6%) and only 1 at grade III (0.7%). No patients stopped protocol therapy because of neurotoxicity, and there was no motor toxicity reported > grade I.

Hypersensitivity reactions to docetaxel were observed in 11 patients (8%), and 4 (3%) were classified as severe. All but 1 patient experienced this reaction on the first or second cycle, and five patients were withdrawn from protocol therapy and continued treatment with single agent carboplatin. All other patients were retreated, with slight schedule modification and further dexamethasone premedication.

Fluid retention was not a significant clinical problem. Increased weight gain or mild peripheral oedema that did not require diuretic therapy was reported for 15 patients (11%).

 *© 2001 Cancer Research Campaign*

**Table 5** Non-haematologic toxicity

| | Grade | Cohort 1 (*n* = 32) Col% | *n* | Cohort 2 (*n* = 22) Col% | *n* | Cohort 3 (*n* = 28) Col% | *n* | Cohort 4 (*n* = 27) Col% | *n* | Cohort 5 (*n* = 30) Col% | *n* |
|---|---|---|---|---|---|---|---|---|---|---|---|
| Nausea | 3 | 13% | 4 | 5% | 1 | 4% | 1 | 7% | 2 | 0% | 0 |
| Vomiting | 3 | 3% | 1 | 5% | 1 | 7% | 2 | 7% | 2 | 0% | 0 |
| Diarrhoea | 3 | 0% | 0 | 0% | 0 | 0% | 0 | 0% | 0 | 7% | 2 |
| | 4 | 3% | 1 | 0% | 0 | 0% | 0 | 4% | 1 | 0% | 0 |
| Stomatitis | 3 | 0% | 0 | 0% | 0 | 0% | 0 | 4% | 1 | 0% | 0 |
| Abdominal pain | 3 | 6% | 2 | 0% | 0 | 7% | 2 | 0% | 0 | 0% | 0 |
| | 4 | 0% | 0 | 0% | 0 | 4% | 1 | 0% | 0 | 0% | 0 |
| Constipation | 3 | 0% | 0 | 5% | 1 | 0% | 0 | 0% | 0 | 10% | 3 |
| Tiredness/fatigue/lethargy | 3 | 0% | 0 | 5% | 1 | 0% | 0 | 7% | 2 | 7% | 2 |
| Sensory neuropathy | 1 | 13% | 4 | 23% | 5 | 36% | 10 | 19% | 5 | 13% | 4 |
| | 2 | 3% | 1 | 9% | 2 | 4% | 1 | 7% | 2 | 3% | 1 |
| | 3 | 0% | 0 | 0% | 0 | 4% | 1 | 0% | 0 | 0% | 0 |
| Motor | 1 | 3% | 1 | 5% | 1 | 0% | 0 | 0% | 0 | 0% | 0 |

**Table 6** Response

| | Cohort 1 Col% | Cohort 1 Count | Cohort 2 Col% | Cohort 2 Count | Cohort 3 Col% | Cohort 3 Count | Cohort 4 Col% | Cohort 4 Count | Cohort 5 Col% | Cohort 5 Count |
|---|---|---|---|---|---|---|---|---|---|---|
| CR | 23% | 5 | 67% | 8 | 43% | 7 | 31% | 5 | 38% | 6 |
| PR | 29% | 6 | 0% | 0 | 25% | 4 | 19% | 3 | 31% | 5 |
| Stable | 14% | 3 | 33% | 4 | 13% | 2 | 37% | 6 | 13% | 2 |
| PD | 19% | 4 | 0% | 0 | 6% | 1 | 6% | 1 | 13% | 2 |
| CR+PR | 61% | 11 | 67% | 8 | 78% | 11 | 53% | 8 | 73% | 11 |
| uneval | 14% | 3 | 0% | 0 | 13% | 2 | 6% | 1 | 7% | 1 |

The Maximum Tolerated Dose (MTD) was reached in both cohorts 4 and 5. In both these cohorts, 2 of the first 6 patients experienced dose-limiting toxicities; in cohort 4, grade IV diarrhoea and a toxic death occurred; in cohort 5 prolonged grade IV neutropenia and grade III diarrhoea were reported.

### Response and survival

73 patients were evaluable for clinical or radiological response at baseline, and 93 were assessable for CA125 response. These results are presented in Table 6. The overall response rate was 66% (49/74) with a 95% confidence interval of 54–77%. 75% (70/93) patients had a CA125 response. Only 9/139 (6%) patients progressed on chemotherapy, stopping treatment before the planned 6 cycles. Progression-free survival for the group as a whole is presented in Figure 1. There is no significant difference in PFS between the groups. Median follow-up for living patients is 19 months (minimum 3 months, maximum 30 months). Median progression-free survival is 16.6 months (95% confidence interval 13.2–19.9 months), and the survival rate at 1 year is 84% (SE = 3%).

### DISCUSSION

This study describes the first experience of docetaxel and carboplatin in combination as the first-line chemotherapy of epithelial ovarian cancer. Although not a randomized trial, 139 eligible chemo-naïve patients were treated with this combination, and this allows some conclusions to be made with regards to its potential utility and acceptability, particularly bearing in mind previous experience with paclitaxel and carboplatin.

The most notable feature of this feasibility study was the extremely low incidence of clinically significant neurotoxicity, especially with carboplatin AUCs up to 7 in combination with docetaxel. No patients were removed from protocol therapy as a direct result of this side-effect, and troublesome functional neuronal impairment – CTC grade II/III toxicity – was reported in less than 6% of all patients. Peripheral sensorineural and motor toxicity is the principal non-myelogenous toxicity of paclitaxel, occurring in up to 80% of patients (Kunitoh et al, 1998), and is dependent upon the cumulative dose and schedule of administration (Rowinski et al, 1993). As a single agent, docetaxel produces neuropathy in only 11% of treated patients (New et al, 1996), and unlike paclitaxel neuropathy, which may manifest early during treatment, docetaxel-induced neuropathy generally does not appear until cumulative doses of docetaxel exceeding 600 mg/m$^2$ (Hilkens et al, 1996). The aetiology of taxane-induced neuronal damage is not completely understood, but is thought to be an effect on neuronal and Schwann cell microtubules with subsequent axonal degeneration and demyelination. It is not clear why docetaxel and paclitaxel differ in the degree of neurotoxicity produced at otherwise equitoxic doses. However, this study clearly shows a lower rate of neuropathy than observed in studies of carboplatin in combination with paclitaxel (Neijt et al, 1996; duBois et al, 1999; Ozols et al, 1999), and suggests a toxicity advantage for docetaxel plus carboplatin, which may be important for longer treatment durations.

General tolerance to the carboplatin-docetaxel combination was excellent, as can be evidenced by Table 5, and the high overall treatment completion rate (Table 1). There were no treatment withdrawals due to fluid retention, confirming that this earlier form of

*© 2001 Cancer Research Campaign* 

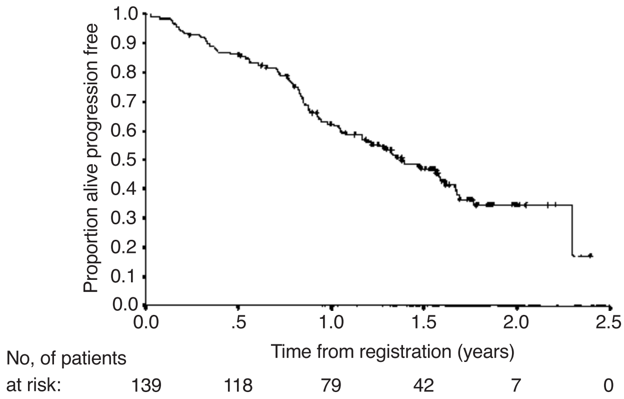

**Figure 1** Progression-free survival, all cohorts Seventy-seven patients have progressed or died (62 are censored)

dose-limiting toxicity can be abrogated successfully by a short, 3-day course of dexamethasone (Docetaxel Investigator's Brochure, 1999). There were no notable, consistent side-effects due to the dose and duration of corticosteroids, and in addition, there was no requirement for routine, prophylactic, intravenous antihypersensitivity medication. Severe hypersensitivity was documented in only 3% of patients, a figure comparable to that documented for docetaxel given as a single agent.

CTC grade III or IV neutropenia occurred in 90% of all patients over the 5 treatment cohorts. That this level of myelosuppression was not accompanied by a higher rate of sepsis is attributable to the generally short duration of neutropenia and lack of significant, accompanying toxicities such as mucositis or diarrhoea, which act as reservoirs for bacterial infection. However, it must be noted that there was at least one (and possibly another) drug-related fatality. Because of the dose-limiting toxicities experienced by patients in cohorts 4 and 5, cohort 2 (carboplatin AUC 5 and docetaxel 75 mg/m$^2$) is thought to represent the best combination, offering a balance between efficacy and toxicity. At these doses, grade III or IV neutropenia was observed in 86% of treated patients (19/22). This is only slightly more than we have observed with the combination of a 3 hour 175 mg/m$^2$ paclitaxel infusion and carboplatin AUC 5, which produces grade III or IV neutropenia in approximately 75% patients (author, unpublished data from SCOTROC trial). The incidence of myelosuppression in this trial with paclitaxel-carboplatin is higher than most reports, but probably reflects the fact that these patients all had weekly haematology performed.

Efficacy and survival were not primary endpoints for this study, as phase II-type data such as this is not directly comparable with survival data obtained from prospective, randomized trials. However, the low rate of on-study progressive disease and a median progression-free survival of greater than 16 months for this non-selected patient population is indicative of significant activity, at least comparable to similar data from early trials with paclitaxel-platinum combinations using both cisplatin and carboplatin. However, in order to truly determine whether the combination of docetaxel and carboplatin can be recommended as routine first-line therapy for epithelial ovarian cancer, prospective randomized comparison with paclitaxel and carboplatin is warranted. In October 1998, the first patient was entered into the SCOTROC Trial (Scottish Randomised Trial in Ovarian Cancer), which randomizes patients with stage Ic–IV ovarian or primary peritoneal cancers to receive either docetaxel or paclitaxel in combination with carboplatin. This international study completed recruitment in May 2000 with 1077 patients randomized, and first survival and toxicity results will be available in May 2001.

Further studies are in progress looking at the possibility of combining other, non-cross resistant agents with carboplatin-docetaxel. There is evidence from two meta-analyses for the additional benefit of incorporating an anthracycline into combination chemotherapy regimens for advanced ovarian cancer. 4 randomized trials have compared an anthracycline-containing regimen ('CAP') with a non-anthracycline containing regimen ('CP'). All these trials demonstrated a slight advantage, albeit not achieving statistical significance, for CAP with regards to survival. However, more recently, a published overview of 2 large meta-analyses (from the AOCTG and OCMP groups) using data from >1700 untreated patients demonstrated that the addition of the anthracycline significantly improved survival (HR 0.85, $P = 0.003$) (A'hern and Gore, 1995). The most common anthracycline in current usage is doxorubicin (Adriamycin), however, epirubicin (Pharmorubicin) is known to have essentially the same spectrum of activity, with less cardiotoxicity, and therefore has a more favourable toxicity profile. Furthermore, epirubicin 60 mg/m$^2$ has been added to the combination of paclitaxel 175 mg/m$^2$ and Carboplatin AUC 7, with a high response rate and manageable toxicities (Hill et al, 1997). In addition, the AGO group have now completed a randomized trial comparing carboplatin-paclitaxel with carboplatin-paclitaxel-epirubicin as first line treatment. The addition of epirubicin to carboplatin-docetaxel as first-line chemotherapy was the subject of a recently completed feasibility study (Vasey et al, 1999 and manuscript in preparation). Utilizing epirubicin doses of 50–60 mg/m$^2$ in combination on day 1 of a 3 week cycle proved to be very myelosuppressive, with cycle delays and dose reductions required in most patients. The recommended dose for this combination is carboplatin reduced to AUC 4 plus docetaxel 75 mg/m$^2$ and epirubicin 50 mg/m$^2$.

In conclusion, this dose-finding study has demonstrated that docetaxel and carboplatin can be combined safely and with significant efficacy as first line chemotherapy for advanced epithelial ovarian cancer. Doses recommended are carboplatin AUC 5 (if glomerular filtration rate measured by $^{51}$CrEDTA) or 6 (if calculated using the Cockcroft-Gault formula) in combination with docetaxel 75 mg/m$^2$. Although myelosuppression is commonly observed, sepsis is rare and neither prophylactic antibiotics nor growth factors are required routinely. Moreover, the incidence of significant neuropathy for patients treated with docetaxel-carboplatin is very low, and may confer a significant toxicity advantage over paclitaxel-carboplatin for this patient population.

### ACKNOWLEDGEMENTS

The authors would also like to thank the following clinicians who also contributed to this study; Drs J Davis and M Deeney, Stobhill Hospital, Springburn, Glasgow; Dr I Duncan, Ninewells Hospital and Medical School, Dundee; Drs EJ Junor, RP Symonds (now at Leicester Royal Infirmary, Leicester) and H Yosef, Beatson Oncology Centre, Glasgow; Dr PG Johnston, Belfast City Hospital, Belfast; Drs J Kennedy and M Soukop, Glasgow Royal Infirmary, Glasgow. In addition, we would like to acknowledge the contribution of all the research nurses who helped in the administration of the chemotherapy and in patient follow-up. Finally, we thank the Cancer Research Campaign who fund our Clinical Trials Office, and Rhone-Poulenc Rorer, for financial support.

*© 2001 Cancer Research Campaign*

Supported by a Grant from Aventis Pharma (formerly Rhone-Poulenc Rorer)

### REFERENCES

Aabo K, Adams M, Adnitt P, Alberts DS, Athanazziou A, Barley V, Bell DR, Bianchi U, Bolis G, Brady MF, Brodovsky HS, Bruckner H, Buyse M, Canetta R, Chylak V, Cohen CJ, Colombo N, Conte PF, Crowther D, Edmonson JH, Gennatas C, Gilbey E, Gore M, Guthrie D, Yeap BY, et al (1998) Chemotherapy in advanced ovarian cancer: four systematic meta-analyses of individual patient data from 37 randomised trials. Advanced Ovarian Cancer Trialists' Group. *Br J Cancer* **78**(11): 1479–1487

A'hern RP and Gore ME (1995) Impact of doxorubicin on survival in advanced ovarian cancer. *J Clin Oncol* **13**(3): 726–732

Bolis G, Scarfone G, Villa A, Acerboni S, Siliprandi V and Guarnerio P (1997) A phase I–II trial of fixed-dose carboplatin and escalating paclitaxel in advanced ovarian cancer. *Eur J Cancer* **33**: 592–595

Bookman MA, McGuire WP 3rd, Kilpatrick D, Keenan E, Hogan WM, Johnson SW, O'Dwyer P, Rowinsky E, Gallion HH and Ozols RF (1996) Carboplatin and paclitaxel in ovarian carcinoma: a phase I study of the Gynaecologic Oncology Group. *J Clin Oncol* **14**(6): 1895–1902

Brown R, Hirst GL, Gallagher WM, McIlwrath AJ, Margison GP, van der Zee AG and Anthoney DA (1997) hMLH1 expression and cellular responses of ovarian tumour cells to treatment with cytotoxic anticancer agents. *Oncogene* **15**: 45–52

Cassidy J, Paul J, Soukop M, Habeshaw T, Reed NS, Parkin D and Kaye SB (1998) Clinical trials of nimodipine as a potential neuroprotector in ovarian cancer patients treated with cisplatinum. *Cancer Chemother Pharmacol* **41**: 161–166

Calvert AH, Newell DR, Gumbrell LA, O'Reilly S, Burnell M, Boxall FE, Siddik ZH, Judson IR, Gore ME and Wiltshaw E (1989) Carboplatin dosage: prospective evaluation of a simple formula based on renal function. *J Clin Oncol* **7**: 1748–1756

Calvert AH, Boddy A, Bailey NP, Siddiqui N, Humphreys A, Hughes A, Robson L, Gumbrell L, Thomas H and Chapman F, et al (1995) Carboplatin in combination with paclitaxel in advanced ovarian cancer: dose determination and pharmacokinetic and pharmacodynamic interactions. *Semin Oncol* **22**(5): 91–98 (suppl 12)

Chan S, Friedrichs K, Noel D, Duarte R, Vorobiof D, Pinter T, Yelle L, Alaki M, Murawsky M and Riva A (1997) A randomised phase III study of taxotere (T) versus doxorubicin (D) in patients with metastatic breast cancer who have failed an alkylating containing regimen: preliminary results. *Proc Am Soc Clin Oncol* **16**: 540 (abstr)

Chantler C, Garnett ES, Parsons V and Veall N (1969) Glomerular filtration rate measurement in man by the single injection method using $^{51}$CrEDTA. *J Clin Sci* **37**: 169–190

Chen XQ, Stroun M, Magnenat JL, Nicod LP, Kurt AM, Lyautey J, Lederrey C and Anker P (1996) Microsatellite alterations in plasma DNA of small cell lung cancer patients. *Nature Med* **2**(9): 1033–1035

Docetaxel Investigator's Brochure, Rhone-Poulenc Rorer Recherche-Developpement, Antony cedex, France. Version 8; 1 September, 1999

duBois A, Luck HJ and Baucknecht T (1997) Phase I/II study of the combination of carboplatin and paclitaxel as first-line chemotherapy in patients with advanced epithelial ovarian cancer. *Ann Oncol* **8**: 355–361

duBois A, Lueck HJ, Meier W, Moebus V, Costa SD, Bauknecht T, Richter B, Warm M, Schroeder W, Olbricht S, Nitz U and Jackisch C (1999) Cisplatin/paclitaxel vs carboplatin/paclitaxel in ovarian cancer: Update of an Arbeitsgemeinschaft Gynaekologische Onkologie (AGO) study group trial. *Proc Am Soc Clin Oncol* **18**: 1374 (abstr)

Eatock M, Paul J and Kaye SB (1999) A new tool for assessing chemotherapy-induced neuropathy. *Br J Cancer* **80**: 111 (suppl 2)

Gianni L (1995) Theoretical and practical aspects of paclitaxel scheduling. *Ann Oncol* **6**: 861–863

Hilkens PH, Verweij J, Stoter G, Vecht CJ, van Putten WL and van den Bent MJ (1996) Peripheral neurotoxicity induced by docetaxel. *Neurology* **46**(1): 104–108

Hilkens PH, Pronk LC, Verweij J, Vecht CJ, van Putten WL and van den Bent MJ (1997) Peripheral neuropathy induced by combination chemotherapy of docetaxel and cisplatin. *Br J Cancer* **75**(3): 417–422

Hill M, Macfarlane V, Moore J and Gore ME (1997) Taxane/Platinum/Anthracycline combination therapy in advanced epithelial ovarian cancer. *Semin Oncol* **24**(1): 34–37 (suppl 2)

Huizing MT, van Warmerdam LJ, Rosing H, Schaefers MC, Lai A, Helmerhorst TJ, Veenhof CH, Birkhofer MJ, Rodenhuis S, Beijnen JH and ten Bokkel Huinink WW (1997) Phase I and pharmacologic study of the combination paclitaxel and carboplatin as first-line chemotherapy in stage III and IV ovarian cancer. *J Clin Oncol* **15**: 1953–1964

Kaye SB, Piccart M, Aapro M, Francis P and Kavanagh J (1997) Phase II trials of docetaxel (Taxotere) in advanced ovarian cancer – an updated overview. *Eur J Cancer* **33**(13): 2167–2170

Kavanagh JJ, Winn R, Steger M, Nelson-Taylor T, Edwards K, Rodgers R, James Borst, Kudelka AP, Hu W and Verschraegen CF (1999) Docetaxel for patients with ovarian cancer refractory to paclitaxel, an update. *Proc Am Soc Clin Oncol* **18**: 1423 (abstr)

Kunitoh H, Saijo N, Furuse K, Noda K and Ogawa M (1998) Neuromuscular toxicities of paclitaxel 210 mg/m$^2$ by 3-hour infusion. *Br J Cancer* **77**: 1686–1688

Lhomme C, Kerbrat P, Lejeune C, Guastalla JP, Fumoleau P, Goupil A, Heron JF, Cassin MA, Pruvot I, Soares JA and Chazard M (1996) Carboplatin plus paclitaxel in the first-line treatment of advanced ovarian cancer: preliminary results of a phase I study. *Semin Oncol* **23**: 48–54 (suppl 12)

McGuire WP, Hoskins WJ, Brady MF, Kucera PR, Partridge EE, Look KY, Clarke-Pearson DL and Davidson M (1996) Cyclophosphamide and cisplatin compared with paclitaxel and cisplatin in patients with stage III and stage IV ovarian cancer. *N Engl J Med* **334**: 1–6

Miller AB, Hoogstraten B, Staquet M and Winkler A (1981) Reporting results of cancer treatment. *Cancer* **74**: 207–214

Nawroz H, Koch W, Anker P, Stroun M and Sidransky D (1996) Microsatellite alterations in serum DNA of head and neck cancer patients. *Nature Med* **2**(9): 1035–1037

Neijt JP, Hansen SW, Hansen SW, Sorensen PG, Sessa C, Witteveen PO, Engelholm SA, Stigaard L, Roer O and Lund B (1997) Randomised phase III study in previously untreated epithelial ovarian cancer FIGO stage IIB, IIC, III, IV, comparing paclitaxel-cisplatin and paclitaxel-carboplatin. *Proc Am Soc Clin Oncol* **16**: 1259 (abstr)

New PZ, Jackson CE, Rinaldi D, Burris H and Barohn RJ (1996) Peripheral neuropathy secondary to docetaxel (Taxotere). *Neurology* **46**: 108–111

Omura GA, Brady MF, Homesley HD, Yordan E, Major FJ, Buchsbaum HJ and Park RC (1991) Long-term follow-up and prognostic factor analysis in advanced ovarian carcinoma: The Gynaecologic Oncology Group experience. *J Clin Oncol* **9**(7): 1138–1150

Ozols RF, Bundy BN, Fowler J, Clarke-Pearson D, Mannel R, Hartenback EM and Baergen R (1999) Randomised phase III study of cisplatin (CIS)/paclitaxel (PAC) versus carboplatin (CARBO)/PAC in optimal stage III epithelial ovarian cancer (OC): A Gynaecologic Oncology Group Trial (GOG 158). *Proc Am Soc Clin Oncol* **18**: 1373 (abstr)

Paridaens R, Bruning P, Klijn J, Gamucci T, Biganzoli L, Van Vreckem A, Hoctin Boes G and Piccart M (1997) An EORTC crossover trial comparing single agent Taxol (T) and doxorubicin (D) as first-and second-line chemotherapy in advanced breast cancer (ABC). *Proc Am Soc Clin Oncol* **16**: 539 (abstr)

Piccart MJ, Bertelsen K, James K, Cassidy J, Mangioni C, Simonsen E, Stuart G, Kaye S, Vergote I, Blom R, Grimshaw R, Atkinson RJ, Swenerton KD, Trope C, Nardi M, Kaern J, Tumolo S, Trimmers P, Roy JA, Lhoas F, Lindvall B, Bacon M, Birt A, Andersen JE, Zee B, Paul J, Baron B and Pecorelli S (2000) Randomised intergroup trial of cisplatin-paclitaxel versus csiplatin-cyclophosphamide in women with advanced epithelial ovarian cancer: three-year results. *J Natl Cancer Inst* **92**(9): 699–708

Pitei DL, Watkins PJ, Stevens MJ and Edmonds ME (1994) The value of the neurometer in assessing diabetic neuropathy. *Diabetic Med* **11**: 872

Righetti SC, Della Torre G, Pilotti S, Menard S, Ottone F, Colnaghi MI, Pierotti MA, Lavarino C, Cornarotti M, Oriana S, Bohm S, Bresciani GL, Spatti G and Zunino F (1996) A comparative study of p53 gene mutations, protein accumulation and response to cisplatin-based chemotherapy in advanced ovarian cancer. *Cancer Res* **56**: 689–693

Rowinsky EK, Chaudhry V, Cornblath DR and Donehower RC (1993) Neurotoxicity of Taxol. *J Natl Cancer Inst Monographs*: 107–115

Rustin GJ, Nelstrop AE, McClean P, Brady MF, McGuire WP, Hoskins WJ, Mitchell H and Lambert HE (1996) Defining response of ovarian carcinoma to initial chemotherapy according to serum CA125. *J Clin Oncol* **14**(5): 1545–1551

Siddiqui N, Boddy AV, Thomas HD, Bailey NP, Robson L, Lind MJ and Calvert AH (1997) A clinical and pharmacokinetic study of the combination of carboplatin and paclitaxel for epithelial ovarian cancer. *Br J Cancer* **75**: 287–294

Strathdee G, MacKean MJ, Illand M and Brown R (1999) A role for methylation of the hMLH1 promotor in loss of hMLH1 expression and drug resistance in ovarian cancer. *Oncogene* **18**(14): 2335–2341

ten Bokkel Huinink WW, van Warmerdam LJ, Helmerhorst TJ, Schaefers MC, Beijnen JH and Rodenhuis S (1997) Phase II study of the combination carboplatin and paclitaxel in patients with ovarian cancer. *Ann Oncol* **8**: 351–354

© 2001 Cancer Research Campaign 

Valero V (1996) Treatment of patients resistant to paclitaxel therapy. *Anticancer Drugs* **7**: 17–19 (suppl 2)
Vasey PA, Paul J, Birt A, Junor EJ, Reed NS, Symonds RP, Atkinson R, Graham J, Crawford SM, Coleman R, Thomas H, Davis J, Eggleton SP and Kaye SB (1999a) Docetaxel-cisplatin in combination as first-line chemotherapy for advanced epithelial ovarian cancer. *J Clin Oncol* **17**(7): 2069–2080
Vasey PA, Reed NS, Davis J, Paul J, Fleming D, Yosef H, Eggleton P and Kaye SB (1999b) Dose-finding study of carboplatin-epirubicin-docetaxel in advanced epithelial ovarian cancer (EOC). *Proc Am Soc Clin Oncol* **18**: 1490 (abstr)
Yancik R (1993) Ovarian cancer: age contrasts in incidence, histology, disease stage at diagnosis, and mortality. *Cancer* **71**: 517–523, (suppl 2)

*© 2001 Cancer Research Campaign*
